# The effect of implant surgery experience on the learning curve of a dynamic navigation system: an in vitro study

**DOI:** 10.1186/s12903-023-02792-8

**Published:** 2023-02-13

**Authors:** Zonghe Xu, Lin Zhou, Ming Zheng, Yanjun Lin, Wenxiu Huang, Jiang Chen, Yan Li, Dong Wu

**Affiliations:** 1 Fujian Provincial Engineering Research Center of Oral Biomaterial, Fujian Medical University, Fuzhou, 350001 China; 2 School and Hospital of Stomatology, Fujian Medical University, Fuzhou, 350001 China; 3 Research Center of Dental and Craniofacial Implants, Fujian Medical University, Fuzhou, 350001 China

**Keywords:** Dental implant, Learning curve, Navigation system, Surgical experience, Accuracy

## Abstract

**Background:**

Dynamic navigation systems have a broad application prospect in digital implanting field. This study aimed to explore and compare the dynamic navigation system learning curve of dentists with different implant surgery experience through dental models.

**Methods:**

The nine participants from the same hospital were divided equally into three groups. Group 1 (G1) and Group 2 (G2) were dentists who had more than 5 years of implant surgery experience. G1 also had more than 3 years of experience with dynamic navigation, while G2 had no experience with dynamic navigation. Group 3 (G3) consisted of dentists with no implant surgery experience and no experience with dynamic navigation. Each participant sequentially placed two implants (31 and 36) on dental models according to four practice courses (1–3, 4–6, 7–9, 10–12 exercises). Each dentist completed 1–3, 4–6 exercises in one day, and then 7–9 and 10–12 exercises 7 ± 1 days later. The preparation time, surgery time and related implant accuracy were analyzed.

**Results:**

Three groups placed 216 implants in four practice courses. The regressions for preparation time (F = 10.294, R^2^ = 0.284), coronal deviation (F = 4.117, R^2^ = 0.071), apical deviation (F = 13.016, R^2^ = 0.194) and axial deviation (F = 30.736, R^2^ = 0.363) were statistically significant in G2. The regressions for preparation time (F = 9.544, R^2^ = 0.269), surgery time (F = 45.032, R^2^ = 0.455), apical deviation (F = 4.295, R^2^ = 0.074) and axial deviation (F = 21.656, R^2^ = 0.286) were statistically significant in G3. Regarding preparation and surgery time, differences were found between G1 and G3, G2 and G3. Regarding implant accuracy, differences were found in the first two practice courses between G1 and G3.

**Conclusions:**

The operation process of dynamic navigation system is relatively simple and easy to use. The linear regression analysis showed there is a dynamic navigation learning curve for dentists with or without implant experience and the learning curve of surgery time for dentists with implant experience fluctuates. However, dentists with implant experience learn more efficiently and have a shorter learning curve.

## Background

Accurate placement and orientation during implant surgery can reduce complications and prolong the service life of implants [1, 2]. With the further development of digital implanting technology, more and more dentists are choosing digital implanting technology in clinical practice [3, 4]. Digital implant surgery mainly includes static guide-assisted implant surgery, dynamic navigation-assisted implant surgery and robotic arm-assisted implant surgery [5]. There are certain limitations in the clinical applications of static guide, for example, Increasing patient treatment period due to guide fabrication, affecting implant accuracy because of intraoperative guide displacement, non-visibility of the operative area and difficulties in water cooling [6, 7]. Robotic arm has not been widely used in clinical treatment work, mainly because of large size, complex structure, low level of intelligence, single function and low patient acceptance [8, 9], so it needs further research and development.

The dynamic navigation system can better solve the current problems of static guides and implant robots while ensuring implant accuracy [10]. Of course, several factors affect the accuracy of dynamic navigation in clinical applications, such as the accuracy of image data [11], the precision of calibration and registration [12] and the accuracy of the dynamic navigation system itself [13]. Dentists' familiarity with the hardware and software of the dynamic navigation system directly affects the accuracy of the navigation system [14]. Since Wright first applied the learning curve theory to describe the relationship between practice frequency and skill proficiency in the 1940s [15], more and more researchers have applied the learning curve theory to explore whether medical effect and medical resource consumption improve with the increase of medical service frequency and surgical experience.

Studies have shown [16] that refining the learning curve of medical devices can help improve the quality of medical services. Sun and Block et al. [17–19] showed that the dynamic navigation system learning curve showed a plateau after five training courses and that dentists with different surgical experience had similar implant accuracy after reaching the learning curve plateau. However, few studies have compared whether the learning curve establishment process of the dynamic navigation system is the same for different implant-experienced dentists. Therefore, this study attempted to establish the dynamic navigation learning curve for dentists with different implant experience by performing implanting practice on dental models, based on the variation of implant accuracy, preoperative preparation time and operative time in each exercise. In addition, this study explored whether dentists with no implant experience can perform implant surgery skillfully and accurately with the help of the dynamic navigation system. This study is expected to provide a partial reference for the clinical applications of the dynamic navigation system.

## Methods

This study was an in vitro model study, so the hospital ethics committee waived the ethical approval requirements for this study. This study was a double-blind experiment to minimize bias.

### Recruitment and grouping of participants

Participants were divided into three groups based on implant surgery experience and experience with the dynamic navigation system. Each group recruited three participants. All the 9 dentists recruited worked in the same hospital (School and Hospital of Stomatology, Fujian Medical University), and each dentist was unaware of the grouping basis of their group.

Group 1 (G1): three dentists with more than five years of implant surgery experience and more than three years of experience in using the dynamic navigation system;

Group 2 (G2): three dentists with more than 5 years of implant surgery experience and no experience in using the dynamic navigation system;

Group 3 (G3): three dentists with no implant surgery experience and no experience with the dynamic navigation system.

### Dental models preparation and pre-operative planning

**Fig. 1:**
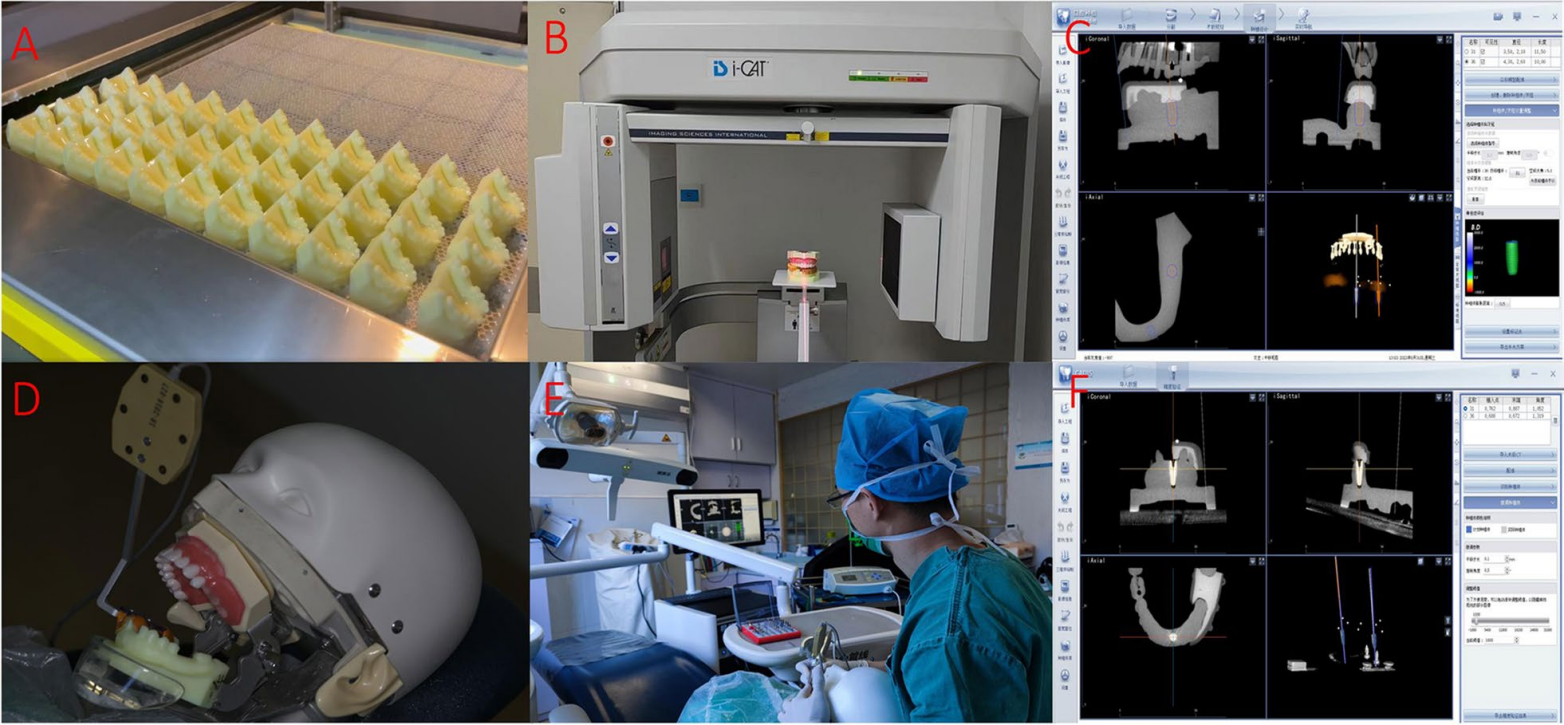
The procedure of the experiment. **A** Printing 3D models **B** Taking preoperative CT. **C** Designing the preoperative plan. **D** Fixing the model and reference plate **E** Implanting practice with the aid of dynamic navigation **F** Postoperative accuracy verification

An intraoral scanner (3Shape, Denmark) was used to scan the mandibular whole-tooth oral teaching model (Tuojin, China). The scanned data were imported into Geomagic Studio, version 2013 (Raindrop, USA) in STL format. 31 and 36 teeth of the model were hollowed out and then filled with mesh to simulate a standard alveolar ridge profile. 108 identical mandibular models were 3D printed (Wanxiang 3D, China) with photosensitive resin (Anycubic, China) (Fig. 1A). One mandibular model was randomly selected, and a suitable registration device (Dcarer, China) was stabilized in the mandibular model using silicone rubber (3M, USA) for preoperative CBCT taking (Fig. 1B). The i-CAT FLX V10 (KaVo, Germany) was used, and the parameters were voltage 120 kV, tube current 5.0 uA, focus 0.5 mm, and voxel size 0.2 mm. All CBCTs were performed by the same physician with extensive clinical experience in dental radiographs.

The DICOM format file of the preoperative CBCT was imported into the oral implant surgery navigation system software (Dcarer, China). According to the three-dimensional information of the dental model, two senior dentists with more than 20 years of implant surgery experience virtually placed two implants in the edentulous areas of 31 and 36 [31: Nobel PMC 3.5 × 11.5 mm; 36: Nobel PMC 4.3 × 10 mm (Nobel, Sweden)]. Through joint consultation, the two senior dentists planned a reasonable preoperative implant planning which was in line with the actual clinical situation (Fig. 1C).

### Implanting practice on dental models

Before the first exercise on dental models, the same experimenter explained the overall procedure of the experiment in detail to each dentist. Dentists who had no experience with the dynamic navigation system watched the same video presentation on how to use the dynamic navigation system. Each dentist's implanting exercise was carried out in the same dental office.

The specific procedure for implanting practice on dental models:

1. The operator stabilized the simulation head model (Tuojin, China) in a dental chair before exercise and adjusted the navigator (Dcarer, China) to position it approximately 1.25–1.5 m from the front of the simulation head model.
2. The handpiece (NSK, Japan) and reference plate (Dcarer, China) were calibrated by infrared tracking sensors (NDI, Canada) to identify the position of the handpiece during the surgery. After calibration, a fixation device (Dcarer, China) fixed the reference plate to the mandibular model. In order to unify the preoperative CBCT, models and surgical instruments into the same space coordinate system, the registration device was completely reset to the edentulous area to register the model and CBCT data (Fig. 1D).
3. The navigation interface showed the positional relationship between the handpiece and the mandibular model based on preoperative planning. Under the guidance of the dynamic navigation system, dentists selectively observed the surgery area in real-time regarding the site, direction, and depth through the dynamic and static views in the navigation interface and adjusted the handpiece position in time. The green interface indicated that the current position is fine, while the red interface indicated that the current position deviates from the designed position. The navigation alert thresholds were set to 0.3 mm for displacement deviation, 3° for angle deviation, and 0.5 mm for depth deviation (Fig. 1E).
4. After implant placement, a postoperative CBCT was taken of the mandibular model.
5. The mandibular model was sorted in the order of 1–12, and each dentist placed two implants in 31 and 36 of the mandibular models according to the order of models. All participants used the drills in the same order for each exercise. According to the same preoperative planning, each dentist repeated 12 exercises on the same mandibular model according to the number of 1–12. Each dentist completed 1–3 exercises in the morning and 4–6 exercises in the afternoon during one day, and 7 ± 1 days later completed 7–9 exercises and 10–12 exercises in the morning and afternoon of a day, respectively.

### Evaluation indices

Preparation time and surgery time: a stopwatch (CASIO, Japan) was used to record the preparation time for preoperative calibration and registration of the dynamic navigation system and the operation time of each implant for each dentist in each exercise. The starting and ending time of the preparation time is from the beginning of calibration to the end of registration. The surgery time is from the dentist to drill according to the dynamic navigation prompt to the dynamic navigation indicates the successful placement of the implant.

Accuracy analysis: the preoperative design scheme and postoperative CBCT data were imported into oral implant surgery accuracy verification software (Dcarer, China). Six special points and surfaces were selected for the preoperative and postoperative rough and fine registration of the model, with an average error of < 0.03 mm (Fig. 1F). The same operator measured the 3D position deviation between the postoperative implant and the preoperative implant.

**Fig. 2:**
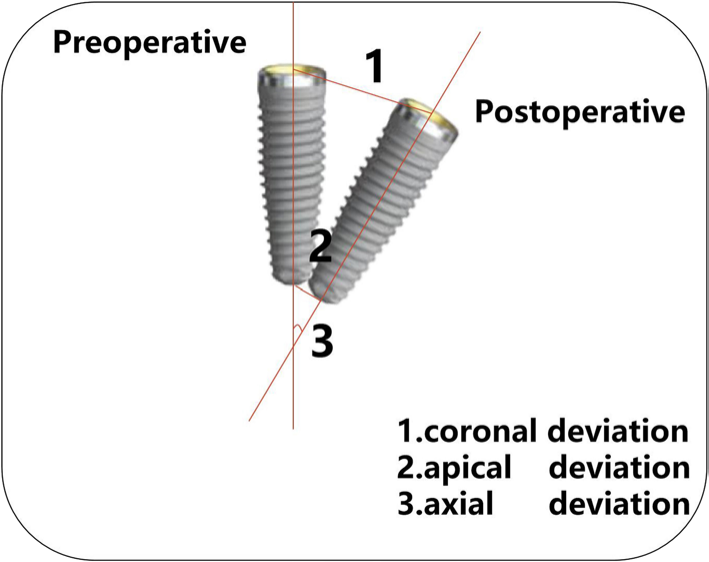
The schematic diagram of accuracy verification

The evaluation indices were as follows (Fig. 2):

1. Coronal Deviation: the linear displacement between the postoperative implant and the preoperative implant at the center of the neck platform of the implant (mm).
2. Apical Deviation: the linear displacement between the postoperative implant and the preoperative implant at the center of the apical part of the implant (mm).
3. Axial Deviation: the intersection angle between the hypothetical central axis of the postoperative implant and the preoperative implant (°).

Preparation time, surgery time and accuracy analysis were performed by the same experimenter who did not know the group of each dentist. The accuracy analysis results were the average of three consecutive repeated measurements.

### Statistical analysis

SPSS 26.0 software (SPAA Inc., USA) was used for statistical analysis and calculated the linear regression equation. Measurement data were expressed as Mean ± SD. Shapiro–Wilk was used to test the normality of data in each group. One-way ANOVA was used to compare the means among the three groups. GraphPad Prism8 (GrapaPad Inc., USA) was used to draw a line chart. *P* value < 0.05 were defined as statistically significant. All statistical analyses were performed by the same experimenter.

## Results

Three groups placed a total of 216 implants. The 12 exercises were divided into 4 courses according to 1–3, 4–6, 7–9 and 10–12 exercises. For each tooth position (31, 36), the two statistical data with the largest deviation from the mean value in each practice course of each group were removed, respectively. The coronal deviation, apical deviation and axial deviation of the 72 implants in G1 were 0.91 ± 0.32 mm, 1.00 ± 0.27 mm and 1.95° ± 0.84°, respectively; the coronal deviation, apical deviation and axial deviation of the 72 implants in G2 were 0.81 ± 0.31 mm, 1.00 ± 0.32 mm and 2.00° ± 0.95°, respectively; the coronal deviation, apical deviation and axial deviation of the 72 implants in G3 were 0.89 ± 0.29 mm, 1.11 ± 0.31 mm and 2.64° ± 0.95°, respectively (Table 1). The preparation time, surgery time, coronal deviation, apical deviation, and axial deviation of the three groups at four courses are summarized in Tables 2 and 3.

**Table 1:** The coronal, apical and axial deviation of 12 exercises for three groups (Mean ± SD)

Group	Number of exercises (times)	Number of implants (pcs)	Coronal deviation (mm)	Apical deviation (mm)	Axial deviation (°)	*p* value^a^	Multiple mean comparisons^b^
Group 1	12	72	0.91 ± 0.32	1.00 ± 0.27	1.95 ± 0.84	< 0.001	coronal deviation < axial deviation apical deviation < axial deviation
Group 2	12	72	0.81 ± 0.31	1.00 ± 0.32	2.00 ± 0.95	< 0.001	coronal deviation < apical deviation coronal deviation < axial deviation apical deviation < axial deviation
Group 3	12	72	0.89 ± 0.29	1.11 ± 0.31	2.64 ± 0.95	< 0.001	coronal deviation < apical deviation coronal deviation < axial deviation apical deviation < axial deviation

^a^One-way ANOVA (*p* < 0.05)

^b^Bonferroni method was used to compare the multiple means for homogeneity of variance; Multiple mean comparisons were performed using Tamhane's T2 test for heterogeneity of variance

**Table 2:** The coronal, apical and axial deviation of four practice courses for three groups (Mean ± SD)

Numbe of exercises (times)	Coronal deviation (mm)				Apical deviation (mm)					Axial deviation (°)				
	Group 1	Group 2	Group 3	*P* value^a^	Group 1	Group 2	Group 3	*p* value^a^	Multiple mean comparisons^b^	Group 1	Group 2	Group 3	*p* value^a^	Multiple mean comparisons^b^
1–3 times	1.08 ± 0.37	0.95 ± 0.40	0.99 ± 0.28	0.671	1.05 ± 0.30	1.24 ± 0.27	1.23 ± 0.36	0.233	NS	2.16 ± 0.93	2.94 ± 0.76	3.30 ± 0.92	0.007	Group 1 < Group 3
4–6 times	0.76 ± 0.28	0.81 ± 0.29	0.89 ± 0.31	0.523	0.91 ± 0.18	0.97 ± 0.31	1.09 ± 0.35	0.313	NS	1.63 ± 0.83	1.97 ± 0.74	2.87 ± 0.66	< 0.001	Group 1 < Group 3 Group 2 < Group 3
7–9 times	0.90 ± 0.31	0.73 ± 0.11	0.91 ± 0.27	0.110	0.99 ± 0.26	0.92 ± 0.15	1.17 ± 0.22	0.015	Group 2 < Group 3	2.04 ± 0.83	1.74 ± 0.54	2.48 ± 0.93	0.068	NS
10–12 times	0.90 ± 0.19	0.73 ± 0.32	0.77 ± 0.25	0.244	1.04 ± 0.27	0.85 ± 0.33	0.95 ± 0.19	0.202	NS	1.96 ± 0.59	1.33 ± 0.84	1.93 ± 0.59	0.089	NS
*p* value^a^	0.070	0.263	0.232		0.520	0.006	0.091			0.392	< 0.001	< 0.001		
Multiple mean comparisons^b^	NS	NS	NS		NS	1–3 times > 7–9 times 1–3 times > 10–12 times	NS			NS	1–3 times > 4–6 times 1–3 times > 7–9 times 1–3 times > 10–12 times	1–3 times > 10–12 times 4–6 times > 10–12 times		

^a^One-way ANOVA (*p* < 0.05)

^b^Bonferroni method was used to compare the multiple means for homogeneity of variance; Multiple mean comparisons were performed using Tamhane's T2 test for heterogeneity of variance

**Table 3:** The preparation and surgery time of four practice courses for three groups (Mean ± SD)

Number of exercises (times)	Preparation time (min)					Surgery time (min)				
	Group 1	Group 2	Group3	*P* value^a^	Multiple mean comparisons^b^	Group 1	Group 2	Group 3	*p* value^a^	Multiple mean comparisons^b^
1–3 times	2.96 ± 0.19	2.99 ± 0.27	3.54 ± 0.38	0.004	Group 1 < Group 3 Group 2 < Group 3	4.87 ± 0.66	4.67 ± 0.86	6.96 ± 0.72	< 0.001	Group 1 < Group 3 Group 2 < Group 3
4–6 times	2.86 ± 0.20	2.79 ± 0.29	3.24 ± 0.35	0.029	Group 2 < Group 3	4.15 ± 0.45	3.80 ± 0.55	6.10 ± 0.51	< 0.001	Group 1 < Group 3 Group 2 < Group 3
7–9 times	2.78 ± 0.10	2.62 ± 0.13	3.09 ± 0.25	0.001	Group 1 < Group 3 Group 2 < Group 3	3.93 ± 0.56	4.25 ± 0.96	5.61 ± 0.82	< 0.001	Group 1 < Group 3 Group 2 < Group 3
10–12 times	2.76 ± 0.14	2.62 ± 0.13	3.04 ± 0.16	< 0.001	Group 1 < Group 3 Group 2 < Group 3	3.63 ± 0.58	4.22 ± 0.88	5.20 ± 0.69	< 0.001	Group 1 < Group 3 Group 2 < Group 3
*p* value^a^	0.143	0.022	0.034			< 0.001	0.029	< 0.001		
Multiple mean comparisons^b^	NS	1–3 times > 7–9 times 1–3 times > 10–12 times	1–3 times > 10–12 times			1–3 times > 4–6 times 1–3 times > 7–9 times 1–3 times > 10–12 times	NS	1–3 times > 4–6 times 1–3 times > 7–9 times 1–3 times > 10–12 times 4–6 times > 10–12 times		

^a^One-way ANOVA (*p* < 0.05)

^b^Bonferroni method was used to compare the multiple means for homogeneity of variance; Multiple mean comparisons were performed using Tamhane's T2 test for heterogeneity of variance

### Preparation time

**Fig. 3:**
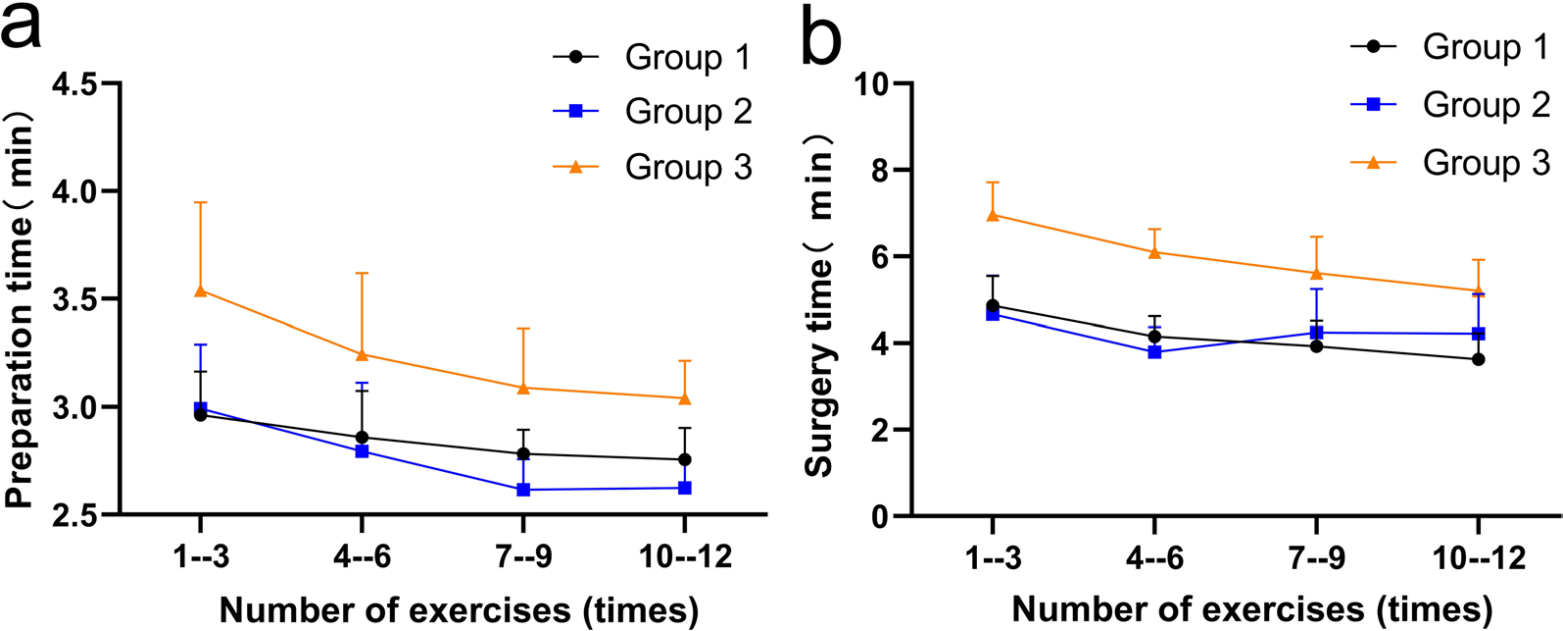
The preparation time (**a**) and surgery time (**b**) of four practice courses for three groups

1. The preparation time of the three groups showed a decreasing trend in the practice process (Fig. 3). Linear regression analysis showed that the preparation time in G1 (F = 5.982, *p* = 0.022), G2 (F = 10.294, *p* = 0.005) and G3 (F = 9.544, *p* = 0.005) were all negatively correlated with practice courses. The regression equation for G1 was preparation time = − 0.70 × practice course + 3.014, R^2^ = 0.187; the regression equation for G2 was preparation time = − 0.128 × practice course + 3.078, R^2^ = 0.284; the regression equation for G3 was preparation time = − 0.165 × practice course + 3.640, R^2^ = 0.269. The preparation time for G2 was shorter than 1–3 exercises at 7–9 exercises (*p* = 0.042) and 10–12 exercises (*p* = 0.049); the preparation time for G3 was less than 1–3 exercises at 10–12 exercises (*p* = 0.048) (Table 3).
2. The preparation time for G2 was shorter than the corresponding preparation time for G3 at 1–3 (*p* = 0.013), 4–6 (*p* = 0.042), 7–9 (*p* = 0.001) and 10–12 (*P* < 0.001) exercises. G1 had less preparation time in 1–3 (*p* = 0.009), 7–9 (*p* = 0.022) and 10–12 (*p* = 0.008) exercises than the corresponding preparation time in G3. The preparation time for each course of G2 was not statistically different from the corresponding preparation time for G1 (Table 3).

### Surgery time

1. The surgery time in G1 and G3 showed a decreasing trend during the practice process (Fig. 3), and linear regression analysis showed that the surgery time in G1 (F = 31.158, *p* < 0.001) and G3 (F = 45.032, *p* < 0.001) was negatively correlated with practice courses. The regression equation for G1 was surgery time = − 0.394 × practice course + 5.129, R^2^ = 0.366; the regression equation for G3 was surgery time = − 0.577 × practice course + 7.409, R^2^ = 0.455. The surgery time for G2 fluctuated during the practice process (Fig. 3), and the regression of surgery time (F = 0.703, *p* = 0.405) was not statistically significant. The surgery time in G1 was less than 1–3 exercises at 4–6 exercises (*p* = 0.014), 7–9 exercises (*p* = 0.001) and 10–12 exercises (*p* < 0.001), and in G3, it was also less than 1–3 exercises at 4–6 exercises (*p* = 0.016), 7–9 exercises (*p* < 0.001) and 10–12 exercises (*p* < 0.001) (Table 3).
2. G2 had a shorter surgery time at 1–3 (*p* < 0.001), 4–6 (*p* < 0.001), 7–9 (*p* < 0.001) and 10–12 (*p* = 0.004) exercises than the corresponding surgery time in G3. G1 also had a shorter surgery time in 1–3 (*p* < 0.001), 4–6 (*p* < 0.001), 7–9 (*p* < 0.001) and 10–12 (*p* < 0.001) exercises than the corresponding surgery time in G3. The surgery time for each course of G2 was not statistically different from the corresponding surgery time in G1 (Table 3).

### Accuracy

The coronal deviation (G1:* p* < 0.001, G2:* p* < 0.001, G3:* p* < 0.001) and apical deviation (G1:* p* < 0.001, G2:* p* < 0.001, G3:* p* < 0.001) were smaller than the axial deviation for all 12 exercises in three groups. The coronal and apical deviations of 12 exercises in G1 were not statistically different. However, the coronal deviation (G2:* p* = 0.006, G3:* p* = 0.001) of 12 exercises in G2 and G3 was smaller than the apical deviation (Table 1).

### Coronal deviation

**Fig. 4:**
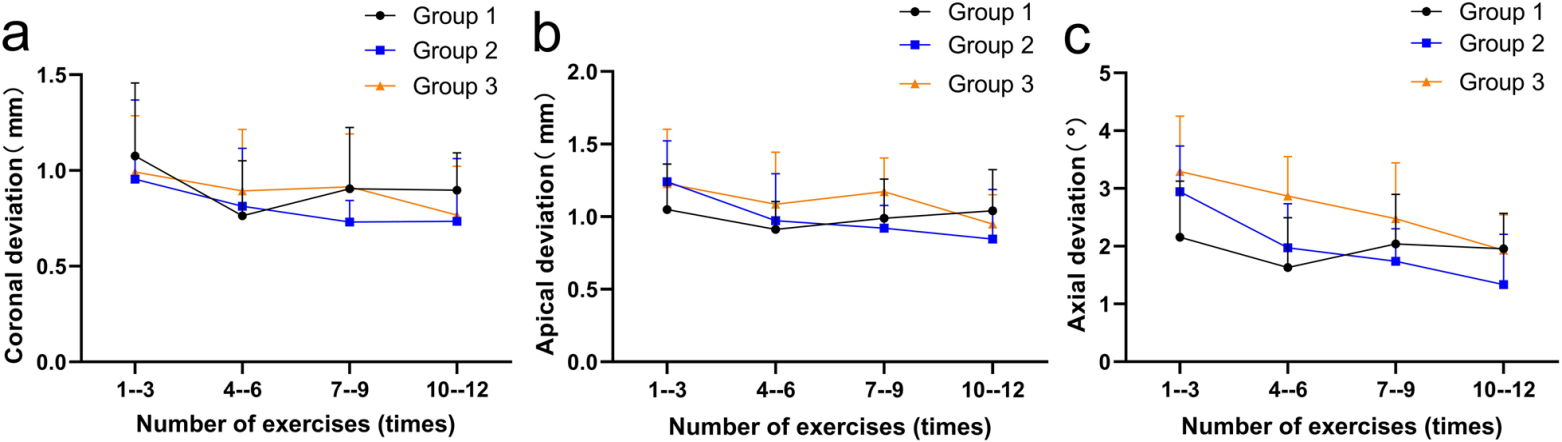
The coronal deviation (**a**), apical deviation (**b**) and axial deviation (**c**) of four practice courses for three groups

1. The coronal deviation in G2 and G3 showed a decreasing trend during the practice process (Fig. 4), and the coronal deviation in G2 was negatively correlated with practice courses (F = 4.117, *p* = 0.047). The regression equation was coronal deviation = − 0.074 × practice course + 0.994, R^2^ = 0.071. The coronal deviation in G1 showed fluctuated (Fig. 4), and the regression of coronal deviation was not statistically significant in G1 (F = 1.106, *p* = 0.298) and G3 (F = 3.701, *p* = 0.060).
2. The corresponding coronal deviation for each practice course in the three groups was not statistically different in a two-by-two comparison (Table 2).

### Apical deviation

1. The apical deviation of G2 and G3 showed a decreasing trend during the practice process (Fig. 4), and the apical deviation of G2 (F = 13.016, *p* = 0.001) and G3 (F = 4.295, *p* = 0.043) were negatively correlated with practice courses. The regression equation for G2 was apical deviation = − 0.123 × practice course + 1.303, R^2^ = 0.194; the regression equation for G3 was apical deviation = − 0.075 × practice course + 1.296, R^2^ = 0.074. The apical deviation for G1 fluctuated during practice process (Fig. 4), and the regression (F = 0.024, *p* = 0.877) was not statistically significant. The apical deviation in G2 was smaller at 7–9 exercises (*p* = 0.008) and 10–12 exercises (*p* = 0.016) than at 1–3 exercises (Table 2).
2. The corresponding apical deviation for each practice course of the three groups was not statistically different when compared two by two, except that the apical deviation at 7–9 exercises (*p* = 0.016) in G2 was smaller than the corresponding apical deviation in G3 (Table 2).

### Axial deviation

1. The axial deviation of G2 and G3 showed a decreasing trend during the practice process (Fig. 4), and the linear regression analysis showed that the axial deviation of G2 (F = 30.736, *p* < 0.001) and G3 (F = 21.656, *p* < 0.001) was negatively correlated with practice courses. The regression equation for G2 was axial deviation = − 0.506 × practice course + 3.262, R^2^ = 0.363. The regression equation for G3 was axial deviation = − 0.448 × practice course + 3.765, R^2^ = 0.286. The axial deviation for G1 fluctuated during the practice process (Fig. 4). The regression of the axial deviation of G1 (F = 1.054, *p* = 0.308) was not statistically significant. The axial deviation in G2 was less than 1–3 exercises at 4–6 exercises (*p* = 0.008), 7–9 exercises (*p* = 0.001) and 10–12 exercises (*p* < 0.001); the axial deviation in G3 was less than 1–3 exercises at 10–12 exercises (*p* < 0.001) (Table 2).
2. The axial deviation of G1 were smaller than the corresponding axial deviation of G3 at 1–3 exercises (*p* = 0.006) and 4–6 exercises (*p* < 0.001). The axial deviation of G2 was smaller than the corresponding axial deviation of G3 at 4–6 exercises (*p* = 0.012). The axial deviation for each practice course in G2 was not statistically different from the corresponding axial deviation in G1 (Table 2).

## Discussion

With the increasingly close integration of computer science, medical radiographs and clinical medicine, digital medicine is growing by leaps and bounds [20, 21]. Increasingly, scholars are focusing on the impact of the learning curve of digital medical devices on the medical service quality. In their systematic review, Arora et al. [22] reported 48 learning curve studies related to cardiothoracic and vascular surgeries. In a systematic review of the learning curve for computer-navigated total knee arthroplasty (TKA), Jenny et al. [23] concluded that navigation may be an effective teaching tool for both beginners and experienced orthopedic surgeons. This study explored the learning curve of dynamic navigation for dentists with different implant experience. It is hoped to provide some help for the clinical applications of dynamic navigation system.

### Preparation and surgery time

Although G1 had more than 3 years of experience in dynamic navigation, the preparation and surgery time in G1 decreased as the number of exercises increased, probably because they performed the same exercises 12 times in a row. G3 had longer preparation and surgery time for each practice course than G2, indicating that dentists with implant experience were more familiar with the whole implant procedure and had better practice results in dynamic navigation preparation and surgery time. As Jenny et al. [23] believed that experienced orthopedic surgeons learned new clinical skills more efficiently and faster than beginners. There was no statistically significant difference in the preparation and surgery time between G2 and G1 in each practice course, suggesting that the dynamic navigator operation logic was relatively simple and easy to use. Even if dentists with implant experience used navigation for the first time, the time spent in preoperative preparation and intraoperative surgery could be accepted. This result is the same as that of Li et al. [24]. Using the first practice course as its control group, we can be found that the preparation time in G2 at 7–9 exercises was statistically different from their preparation time at 1–3 exercises, while in G3 were the ones who showed the difference at 10–12 exercises. This indicated that having certain implant surgery experience can shorten the learning process of dynamic navigation preoperative preparation operation. In terms of surgery time, affected by implant experience, dentists in G2 sometimes drilled according to their own clinical experience and corrected in time under the guidance of dynamic navigation, resulting in certain fluctuations in the learning curve of G2 in terms of surgery time. The linear regression of surgery time with the practice course for G3 was statistically different, consistent with Agha et al.'s findings [25], that students experience a reduction in surgical time as they master a new medical technique. The result of Deeb et al. [26] showed that students experienced a significant improvement in surgery time when practising to place 7–12 implants with the aid of dynamic navigation, which is consistent with what was observed in this study in G3 of surgery time.

### Accuracy

Two previous systematic reviews [27, 28] explored the accuracy of dynamic navigation on in vitro models: The coronal deviation is all 0.91 mm; the apical deviation is 1.21 mm and 1.04 mm, and the axial deviation is 2.78° and 3.7°. This is similar to the accuracy of dentists in G1 of this study [coronal deviation (0.91 ± 0.32 mm), apical deviation (1.00 ± 0.27 mm) and axial deviation (1.95° ± 0.84°)], indicating that the dynamic navigation used in this study has the same level of accuracy as other dynamic navigation. The coronal and apical deviation were smaller than the axial deviation for all three groups during the entire practice, suggesting that dentists should pay attention to axial control when place implants with navigation assistance. In G1, the total coronal deviation was not less than the total apical deviation in 12 exercises as in G2 and G3, indicating that implants can be better controlled with skilled use of dynamic navigation.

The linear regressions of the coronal, apical and axial deviation during the practice process in G1 were not statistically significant. However, the linear regressions of the coronal, apical and axial deviation during the practice process in G2 and the apical and axial deviation during the practice process in G3 were statistically significant. Although the coronal deviation in G3 did not show a statistically significant linear regression during the practice process, it showed a decreasing trend. This suggests that there is a learning curve in dynamic navigation for dentists with or without implant experience, and that repeated use of dynamic navigation several times before reaching the plateau of the learning curve allows dentists to become more proficient and thus improves implant accuracy. Wang et al. [29] also found a learning curve for dynamic navigation among dental students, but they did not study dentists' learning curve. There was no statistical difference in coronal deviation among the three groups at each practice course, and for apical deviation there was no statistical difference among the three groups at each practice course, except for the statistical difference between G2 and G3 at 7–9 exercises. This indicates that the current dynamic navigation operation logic is relatively simple and easy to use and that coronal and apical sites of implants can be controlled during the first practice course, regardless of dentists' implant experience. The study by Sun et al. [19] showed that the learning curve of dynamic navigation will reach a plateau after five training courses. However, this conclusion was not reached in G2. This conclusion is reflected in the axial deviation of G3: The axial deviation of G3 after 4–6 exercises was not statistically different from that of G1. This may be because the dynamic navigation system used in this study has simple operation logic and can be quickly used by dentists. Kan et al. [30] argued that the operating logic of digital devices directly affects the operating process. We can find higher learning efficiency of dentists with implant experience in the axial deviation: axial deviation in G2 was not statistically different from G1 during all practice courses, while G3 required 4–6 exercises. Using the first practice course as their own control group, we found that the apical deviation of G2 at 7–9 exercises was statistically different from their apical deviation at 1–3 exercises, while G3 did not show a difference at 10–12 exercises; the axial deviation of G2 at 4–6 exercises was statistically different from their axial deviation at 1–3 exercises, while G3 showed a difference at 7–9 exercises. This indicates that having certain surgical experience can shorten the learning process of dynamic navigation.

Agachan et al. [31] concluded that it took approximately 50 cases for surgeons to learn laparoscopic colorectal surgery by conventional surgery and that the complication rate decreased significantly as dentists gained experience. In the last two practice courses of this study, there was no statistical difference between the coronal deviation, apical deviation and axial deviation in all three groups, which suggests that after mastering dynamic navigation, implant accuracy is not affected by dentists' own clinical experience, even if the operator is a beginner without implant experience. This was also the conclusion of Wu et al. [32] and Block et al. [33]. This finding proves that the dynamic navigation system can help beginners shorten the learning process in clinical surgery, without slowly reducing the occurrence of surgical complications through a large number of clinical surgeries. Zhan et al. [34] compared dental students' learning progress in dental implant placement between a dynamic navigation system and a traditional training method. They concluded that the accuracy of beginners in the dynamic navigation group was significantly improved compared to the traditional technique group. The experimental results of this study showed that the learning efficiency of dentists was improved with the assistance of dynamic navigation. The possible reason is that dentists received real-time feedback during the operation. When using dynamic navigation for implant surgery, dentists can detect the deviation between the current site and the ideal site in time and solve the problem in real time through the remainder of the dynamic navigation system to realize precise digital implantation and greatly shorten the learning process.

It should be noted that this study used a simulated head model for in vitro practice. Although simulated head models have been widely used in dental teaching [35], current simulated head models cannot adequately simulate the factors such as blood, saliva and soft tissue in the oral environment. In actual clinical surgery, dentists may face other disturbances, such as limited mouth opening, excessive bleeding and patient mobility. Therefore, in vitro practice cannot be fully equated with specific operations in clinical practice. In addition, this study has some limitations: the effects of implantation site or gender of dentists on the experimental results were not considered, and the possible relevant experience information, such as computer game experience, was not counted. The limited number of participants may also lead to bias in the experiment results.

## Conclusions

Despite the limitations of this study, the following conclusions can be drawn.

1. The procedure of the navigation system is relatively simple and easy to master. However, operators must pay attention to the overall grasp of the implant's coronal, apical and axial direction during the operation, especially the axial deviation.
2. There is a dynamic navigation learning curve for dentists with or without implant experience, but there are fluctuations in the dynamic navigation learning curve of surgeon time for dentists with implant experience.
3. Dentists with implant experience learn more efficiently and have a shorter dynamic navigation learning curve. With the assist of dynamic navigation systems, dentists with no implant experience could master the control and apical sites of the implant at 1–3 exercises, improve the surgeon time at 4–6 exercises and reach the axial deviation plateau at 7–9 exercises.

## Data Availability

The data used and analysed during the current study are available from the corresponding author on reasonable request.

## Abbreviations

CBCT: Cone beam computed tomography
CT: Computed tomography
DICOM: Digital imaging and communications in medicine
STL: STereoLithography
